# A Chromosome-Level Genome of ‘Xiaobaixing’ (*Prunus armeniaca* L.) Provides Clues to Its Domestication and Identification of Key *bHLH* Genes in Amygdalin Biosynthesis

**DOI:** 10.3390/plants12152756

**Published:** 2023-07-25

**Authors:** Ling Guo, Fangjie Xie, Xue Huang, Zhengrong Luo

**Affiliations:** 1 National Key Laboratory for Germplasm Innovation & Utilization of Horticultural Crops, Huazhong Agricultural University, Wuhan 430070, China; glzky@163.com; 2 College of Horticulture and Forestry, Tarim University, Alar 843300, China; 17778649365@163.com (F.X.); h1723712462@163.com (X.H.); 3 Xinjiang Production & Construction Corps Key Laboratory of Protection and Utilization of Biological Resources in Tarim Basin, Alar 843300, China

**Keywords:** xiaobaixing, amygdalin, genome, *bHLH*, biosynthesis

## Abstract

Apricot is a widely cultivated fruit tree of the drupe family, and its sweet/bitter kernel traits are important indicators of the quality and merchantability of apricots. The sweetness/bitterness traits were mainly determined by amygdalin content. However, the lack of high-quality genomes has limited insight into the traits. In this study, a high-quality genome of ‘Xiaobaixing’ was obtained by using single-molecule sequencing and chromosome-conformation capture techniques, with eight chromosomes of 0.21 Gb in length and 52.80% repetitive sequences. A total of 29,157 protein-coding genes were predicted with contigs N50 = 3.56 Mb and scaffold N50 = 26.73 Mb. Construction of phylogenetic trees of 15 species of Rosaceae fruit trees, with ‘Xiaobaixing’ differentiated by 5.3 Ma as the closest relative to ‘Yinxiangbai’. GO functional annotation and KEGG enrichment analysis identified 227 specific gene families to ‘Xiaobaixing’, with 569 expansion-gene families and 1316 contraction-gene families, including the significant expansion of phenylalanine N-monooxygenase and β-glucosidase genes associated with amygdalin synthesis, significant contraction of wild black cherry glucoside β-glucosidase genes, amygdalin β-glucosidase genes, and β-glucosidase genes, and significant enrichment of positively selected genes in the cyanogenic amino acid metabolic pathway. The 88 *bHLH* genes were identified in the genome of ‘Xiaobaixing’, and *ParbHLH66* (*rna-Par24659.1*) was found to be a key gene for the identification of sweet/bitter kernels of apricots. The amino acid sequence encoded by its gene is highly conserved in the species of *Prunus mume*, *Prunus dulcis*, *Prunus persica*, and *Prunus avium* and may be participating in the regulation of amygdalin biosynthesis, which provides a theoretical foundation for the molecular identification of sweet/bitter kernels of apricots.

## 1. Introduction

The apricot, a member of the Rosaceae family, is an important fruit with a distinctive flavor and has important economic and nutritional values [1]. Xinjiang is one of the centers of origin of apricots in the world and has rich germplasm resources for cultivated and wild apricots [2]. ‘Xiaobaixing’ is a characteristic locally cultivated apricot of Kuqa County (Aksu, Xinjiang). Both its area and production are the largest in China [3]. The kernels are thin-shelled and sweet and are excellent germplasm for both fruit and kernels [4]. Apricot has rich nutritional value, low amygdalin content, sweet flavor, and high kernel yield [5]. Sweet/bitter traits are important indicators to evaluate the quality and commodity of apricots, which are mainly determined by the amygdalin content [6,7].

Amygdalin, a secondary metabolite of the plant, is the active ingredient in bitter apricot [8], which has important health and medicinal values such as anti-inflammatory, anti-cancer, and anti-tumor activities [9,10]. It is widely found in the kernels of apricots, *P. persica*, *P. mumes*, loquats, and apples [11,12,13], with the most significant content in bitter kernels of apricots [14]. Femenia et al. [15] showed that the content of amygdalin in bitter seed kernels of apricots was very high (5.5 g/100 g). Asma et al. [16] showed that 72.04% of 128 Turkey apricots were sweet kernels, and Krichen et al. [17] found that more than 1/4 had a sweet taste among 112 Tunisian apricots. Wani et al. [18] showed that 57.50% of the kernels were sweet and 42.50% were bitter in 40 Indian apricots. Amygdalin is contained in the range of 0.95 to 36.06 mg/g in 88 germplasm resources of apricots [19]. Previous studies have shown differences in kernels’ economic traits in different geographical environments and the production of bitter substances during the development of both sweet/bitter kernels in apricot [20].

Amygdalin content is not only related to ecology and type [21], but the genotypic selection has a greater effect on amygdalin content [22]. The metabolic pathway of amygdalin is mainly regulated by the phenylalanine metabolic pathway. CYP79D16 and CYP71AN24 in the pathway are cytochrome P450 (CYP) enzymes that catalyze the conversion of phenylalanine (Phe) to mandelonitrile (MAN), which mediates the biosynthesis of bitter amygdalin [23]. The *bHLH* transcription factor is the second-largest transcription factor after *MYB*, and it plays a key regulatory role in plant growth and development, stress resistance, and metabolite synthesis [24,25,26]. Studies have shown that the *bHLH2* gene regulates the transcription of CYP79D16 and CYP71AN24 and thus regulates bitter flavor [27]. Gong et al. [28] found that the *bHLH* gene (*Cla011508*) regulates the bitterness of watermelon fruit in the F2 population of two inbred lines, namely ‘9904’ (bitter) and ‘Handel’ (non-bitter). Xu et al. [29] identified 1160 *bHLH* genes in the Cucurbits genome and found that two tandem *bHLH* genes were involved in the regulation of cucurbitacin biosynthesis in the root system. However, the regulation of sweet/bitter traits in apricot by the *bHLH* gene is unclear.

A small number of apricot genomes have been reported in China and abroad, of which Jiang et al. [30] used *P. armeniaca* (‘Chuanzhihong’) as material and sequenced it using Sequel SMRT Cells published in 2019. The first high-quality reference genome, with a 221.9 Mbp genome size and 92.88% coverage, annotated 30,436 genes with 38.28% repetitive sequences. Yuan et al. [31] have sequenced and assembled ‘Meihua’ apricot using a combination of Illumina, PacBio, and Hi-C technologies, obtaining a genome size of 238.49 Mbp, a genome heterozygosity of 0.425%, repetitive sequences accounting for 47.1%, and predicting 23,445 genes. Zhang et al. obtained high-quality sequences of eight apricot chromosomes with a genome size of 251.19 Mb, a heterozygosity of 0.99%, a duplicate sequence of 46.78%, and 29,230 genes were annotated by using triple-sequencing technology combined with second-generation data error correction and HiC sequencing for sequence mounting using the ‘Yinxiangbai’ apricot as sequencing material [32]. Chen et al. determined and assembled three genomes, namely *P. sibirica* (the F106 genome was 219 Mb in size, with an overlapping cluster N50 length of 6.70 Mb and eight chromosomes, with 32,959 genes predicted), *P. armeniaca* (the genome was 217 Mb, with an overlap cluster N50 length of 7.13 Mb, eight chromosomes, and 32,669 genes were predicted), and *P. armeniaca* × *P. sibirica* (the genome was 225 Mb, with an overlap cluster N50 length of 6.91 Mb, eight chromosomes and 32,987 genes were predicted) and the results of the study were found to be different [33]. The above studies have shown that different germplasm has an effect on the size of the genome assembled, the size of the N50, the size of the pseudomolecules, and the number of predicted genes.

Sweet kernels are the dominant trait in the local apricots, but the scientific aspects of their character and variation, such as the disappearance of bitterness and the enhancement of sweetness, are not yet clear. The amygdalin content of apricots determines their sweetness/bitterness, and its level is an important indicator of quality and merchantability and an important factor in determining the adaptive evolution of apricot types. However, differences in the amygdalin content of different apricot germplasm are necessarily associated with variations in the nucleotide sequences of their genes. However, the apricot germplasm is necessarily related to the nucleotide sequence variation of its genes. The mechanism of amygdalin synthesis of whole genome sequence has not yet been reported, and the lack of high-quality genomes limits insight into the trait. In this study, the whole genome map of apricot was constructed using ‘Xiaobaixing’ in southern Xinjiang as material, and the *bHLH* gene family related to sweet/bitter traits in apricot was identified, which provided a direction for the regulation of amygdalin and contributed to the understanding of the evolutionary process in apricot. The study will also provide a key trait gene and conduct future gene function studies.

## 2. Results

### 2.1. Genome Sequencing and Assembly

**Figure 1:**
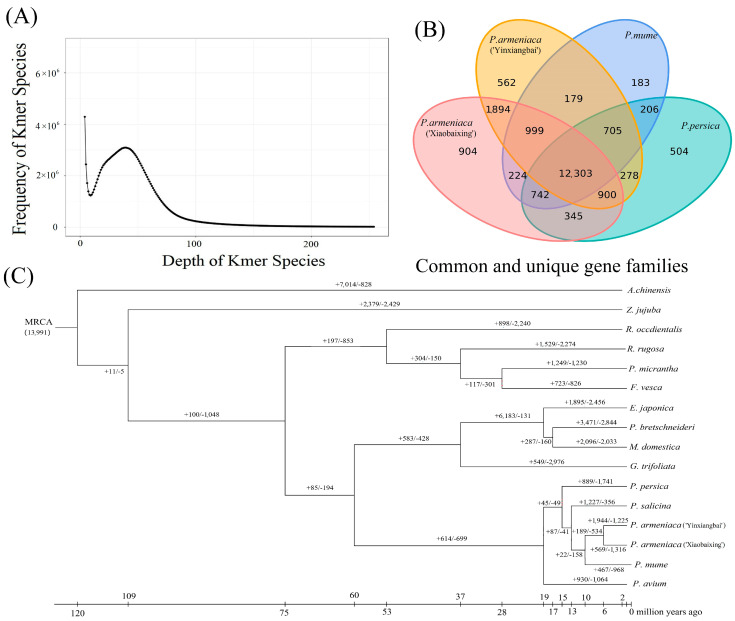
Frequency distribution of K-mer depth and the number of K-mer species. (**A**) K-mer analysis to estimate the ‘Xiaobaixing’ genome size. (**B**) Statistical results of gene families in Xiaobaixing and other species. (**C**) Phylogenetic trees of the 15 plants and the expansion and contraction of their gene families.

The raw data from genome sequencing were quality-controlled to obtain clean data 23,649,264,900 bp (Table S1 and Figure S1), which had normal sequencing quality and error rate. The NT database comparison showed that the data did not contain significant exogenous contamination. K-mer analysis revealed that the sample genome size was approximately 451.72 Mbp, the heterozygosity rate was 0.98%, the proportion of repetitive sequences was 68.94%, and the genomic GC content was approximately 42.05% (Figure 1A and Table S2). Further assembly of the ‘Xiaobaixing’ genome showed that the total contig length was 0.23 Gb, and the contig N50 reached 3.19 Mb (Table S3). The rate of reads compared to the assembled genome was 96.18% and 99.95% for the second and third-generation data, respectively, and the rate of the assembled genome being covered by reads was 99.23% and 99.7%, respectively, indicating a high degree of sequence identity between the reads and the assembled genome (Table S4). The 1552 genes were able to be completely aligned into the genome by BUSCO software, which indicated the high quality of genome assembly in ‘Xiaobaixing’ (Table S5). The original sequence of the apricot genome was 0.23 Gb in length; the initial assembly of contigs N50 = 3.19 Mb, with 433 contigs (Table S6). After Hi-C assisted genome assembly, the final length of the apricot genome that anchored to eight chromosomes (containing 139 contigs) was determined to be 0.21 Gb, contigs N50 = 3.56 Mb, scaffold N50 = 26.73 Mb, contig length anchoring rate was 92.23%, and contig number anchoring rate was 32.10%.

### 2.2. Genome Annotation

Repeated sequences accounted for 52.80% of the genome, with long terminal repeat (LTR) accounting for 37.04% and DNA transposons for 12.16%. De novo prediction was performed using Augustus, GlimmerHMM, GenScan and Gene ID, and then homology prediction was performed in combination with apricot (‘String Red’ and ‘Yinxiangbai’), *Prunus mume* Siebold & Zucc., *Prunus salicina* Lindl. and *Prunus persica* (L.) Batsch). The results indicate that 29,157 protein-coding genes were finally predicted, with an average gene length of 2886.86 bp, an average CDS sequence length of 1036.09 bp, an average of 4.29 exons, and an average exon and intron length of 243.01 bp and 563.53 bp, respectively (Table S7). 2316 genes have not annotated, 92.06% were annotated to InterPro, GO, KEGG ALL, KEGG KO, SwissProt, TrEMBL and NR respectively accounting for 69.12%, 50.48%, 85.20%, 30.71%, 60.83%, 91.76% and 90.55%. The ncRNA information of the apricot genome was obtained by comparing it with the known ncRNA database (Table S8).

### 2.3. Phylogenetic Analysis

The annotated genes of 15 plants, such as *P. armeniaca*, *P. mume*, and *P. persica*, were clustered, and 481,817 genes were obtained, with a total of 149 single-copy genes. Of these, ‘Xiaobaixing’ had a total of 29,157 genes and 227 specific gene families, with an average gene family number of 1.48 genes (Table S9). As shown in Figure 1B, a total of 12,303 gene families were identified in the four comparisons, and 904 gene families were specific to ‘Xiaobaixing’. We further constructed phylogenetic trees, as shown in Figure 1C, indicating that ‘Yinxiangbai’ diverged from ‘Xiaobaixing’ by 5.3 Ma and the apricot diverged from *P. mume* by 9.6 Ma. *P. armeniaca*, *P. salicina*, *P. mume*, *P. persica*, and *P. avium* diverged on a branch from the most recent common ancestor (MRCA) of loquat, *P. bretschneideri*, *M. domestica*, and *G. trifoliata* about 58.4 Ma ago. Comparing the common ancestors of *P. armeniaca* and *P. mume*, 569 expansion-gene families and 1316 contraction-gene families were found in ‘Xiaobaixing’, while ‘Yinxiangbai’ had 1944 expansion-gene families and 1225 contraction-gene families.

### 2.4. KEGG Enrichment and Metabolic Pathway Analysis

**Figure 2:**
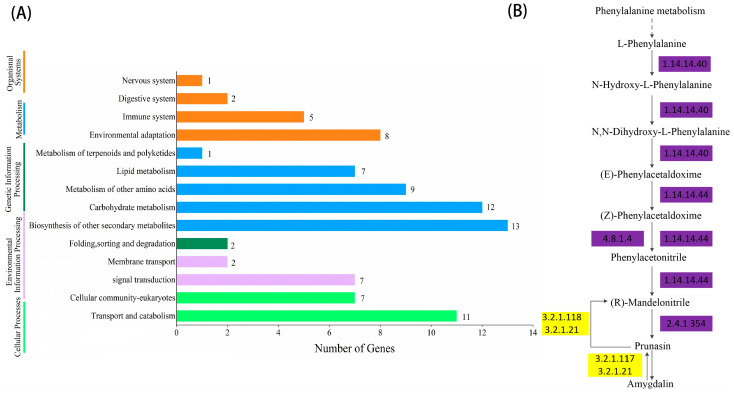
Histogram of KEGG gene classification and metabolic pathway of significantly contracted gene families. (**A**) KEGG gene classification histogram for the significantly contractile gene family. (**B**) Chromosome-level genome-based metabolic pathway.

**Figure 3:**
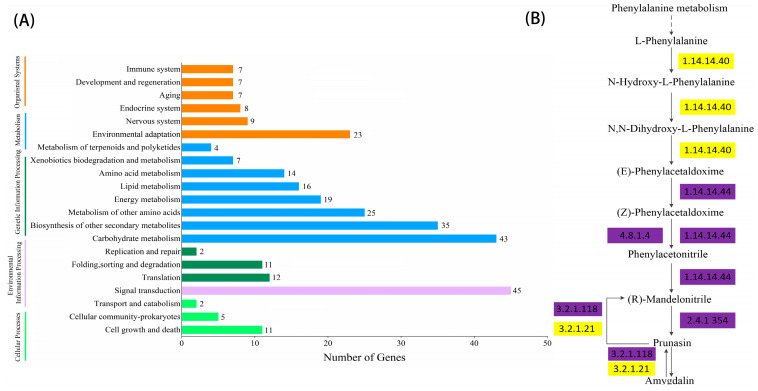
Histogram of KEGG gene classification and metabolic pathway of significantly expansion gene families. (**A**) KEGG gene classification histogram for the significantly expansion gene family. (**B**) Chromosome-level genome-based metabolic pathway.

**Figure 4:**
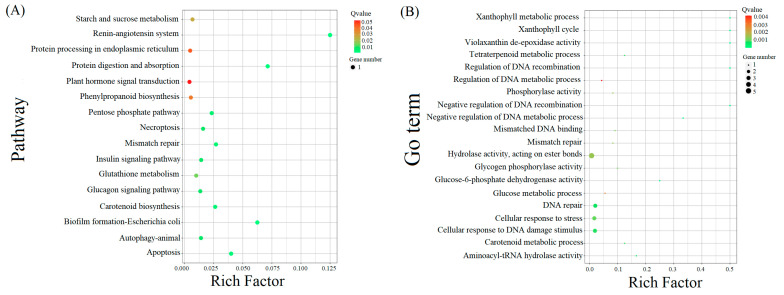
KEGG pathway enrichment of expanded genes and specific in Xiaobaixing. (**A**) KEGG enrichment scatter plot. (**B**) GO enrichment scatter plot.

As shown in Figure 2 and Figure 3, the significant expansion and contraction-gene families were analyzed for KEGG enrichment and combined with metabolic pathway maps. The results revealed that nine genes (β-glucosidase gene, wild black cherry gene, and amygdalin β-glucosidase gene) were significantly contracted, and 18 genes (*CYP79A2* and β-glucosidase gene) were significantly expanded during the synthesis of amygdalin. Further analysis of genes in ‘Xiaobaixing’ showed that 313 genes were significantly contracted and 898 genes were significantly expanded. KEGG-enrichment analysis revealed a significant contraction of genes related to the synthesis of various metabolites such as fatty acids, phenylpropanoids, alcohols, carotenoids, and flavonoids in ‘Xiaobaixing’, with genes related to inner core size still to be explored. A significant expansion of synthetic genes related to various metabolites such as indole alkaloids, α-linolenic acid, linoleic acid, isoquinoline alkaloids, cytochrome P450, triterpenoids, starch, sucrose, steroids, glutathione, ascorbic acid, and aldehydes occurred in ‘Xiaobingxing’ (Table S10). As shown in Figure 4, the significantly positively selected genes were further analyzed for GO and KEGG enrichment. GO functional enrichment revealed that these genes were significantly enriched in several categories related to secondary product metabolism, DNA repair, cellular response to stress, and cellular response to DNA damage stimulation. The KEGG metabolic pathway indicated that positively selected genes were significantly enriched in the cyanogenic amino acid metabolism, DNA replication, and repair pathways.

### 2.5. Collinear Analysis

**Figure 5:**
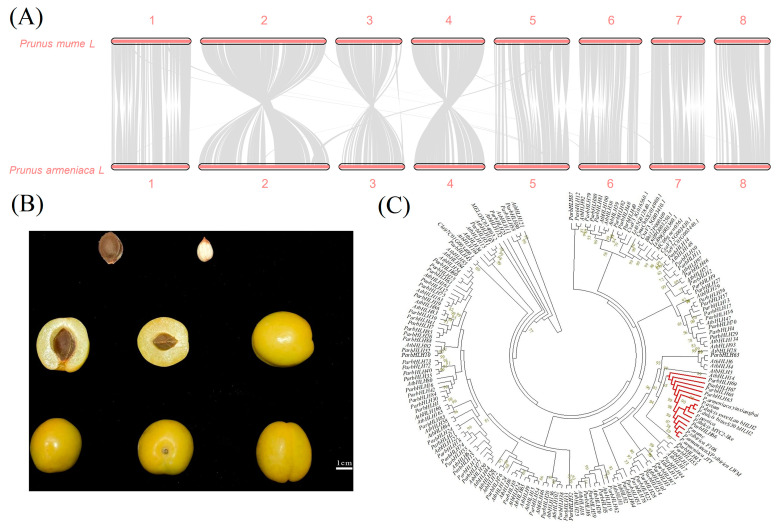
Co-linear analysis and phylogenetic tree analysis of *bHLH* genes in ‘Xiaobaixing’. (**A**) Co-linear analysis comparison results. (**B**) Diagram of ‘Xiaobaixing’ fruit and inner kernels. (**C**) Phylogenetic tree analysis of bHLH proteins among different species.

The assembled apricot genome was compared with the published *P. mume* genome in a two-by-two cycle, as shown in Figure 5A, and generally, the assembled genome showed good co-linearity with the reference genome.

### 2.6. Analysis of the bHLH Gene

#### 2.6.1. The Analysis of the Nucleotide Sequence for the *bHLH* Gene in Apricot

To explore the evolutionary events of putative genes involved in bitterness formation in ‘Xiaobaixing’, a total of 88 *bHLH* family genes were identified in this study from the genome-wide database of ‘Xiaobaixing’. They were named *ParbHLH1*-*ParBHLH88*, of which *ParbHLH66* and *rna-Par24659.1* were the same gene. The nucleotide sequences of the *bHLH* genes were analyzed online by SMS. The nucleotide sequences of the *bHLH* genes were analyzed by SMS, which revealed a large variation in sequence length among the 88 genes, with *ParbHLH8*, *ParbHLH38*, and *ParbHLH36* showing longer sequences, ranging from 2241 to 3168 bp in full length, and *ParbHLH37*, *ParbHLH75*, and *ParbHLH19* showing shorter sequences, ranging from 276 to 285 bp in full length. The proportions of the 88 *bHLH* gene base pairs were relatively different from each other, with the lowest base G + C content of 41.6% and the highest base A + T content of 58.4% for *ParbHLH67*. The highest base G + C content of *ParbHLH28* was 62.2%, while the lowest base A + T content was 37.8% (Table S11).

#### 2.6.2. Analysis of the Amino Acids Encoded by the *bHLH* Gene Family in Apricot

Analysis of the physicochemical properties of the bHLH protein sequence of apricot (Table S12) showed that ParbHLH75 had the lowest relative molecular weight of 10,285.48 Da and ParbHLH36 had the highest relative molecular weight of 116,384.31 Da; the theoretical isoelectric point (*pI*) range was 4.49 to 9.91; ParbHLH36 contained the highest number of negatively charged amino acids. The highest number of negatively charged amino acids in the ParbHLH36 protein was 122, the lowest number of negatively charged amino acids in the ParbHLH75 protein was 12, the highest number of positively charged amino acids in the ParbHLH36 and ParbHLH38 protein was 116, and the lowest number of positively charged amino acids in the ParbHLH75 band was 14. The protein-instability index ranged from 39.97 to 95.36, with the lowest instability index for ParbHLH73 and the highest instability index for ParbHLH75; the lipid-bound protein index ranged from 51.77 to 104.49, with ParbHLH54 the lowest and ParbHLH14 the highest. Most bHLH proteins were composed mainly of serine.

#### 2.6.3. Phylogenetic Analysis

To investigate the evolutionary relationships and functions of bHLH proteins in apricot and to identify proteins involved in bitterness biosynthesis. In this study, a phylogenetic tree was constructed using 88 ParbHLH proteins, AtbHLH proteins, and primers to identify sweet/bitter-related proteins and bHLH proteins that regulate bitterness in Cucurbitaceae (Figure 5B,C). The results showed that ParbHLH66 clustered with the proteins identified by ‘Long Wang Cap’, ‘F106’, ‘Golden Sun’, and the primers used in *P. mume*. It indicates that apricots are highly homologous to *P. mume* genes for regulating sweetness/bitterness. The proteins identified in apricot, *P. mume*, *P. persica*, *P. dulcis*, and *P. avium* using primers were clustered with the *bHLH2* protein associated with amygdalin biosynthesis in *P. dulcis*. It indicated that *ParbHLH66* was a key gene in the regulation of amygdalin. In addition, *ParbHLH66* was found to form a crosstalk repeat gene pair with *ParbHLH65*, *ParbHLH68*, *ParbHLH67*, and *ParbHLH69*. Therefore, it was probable that the gene cluster was involved in the biosynthesis of bitter amygdalin in ‘Xiaobaixing’.

#### 2.6.4. Analysis of the *bHLH* Family Gene Structure and Conserved Motifs in ‘Xiaobaixing’

**Figure 6:**
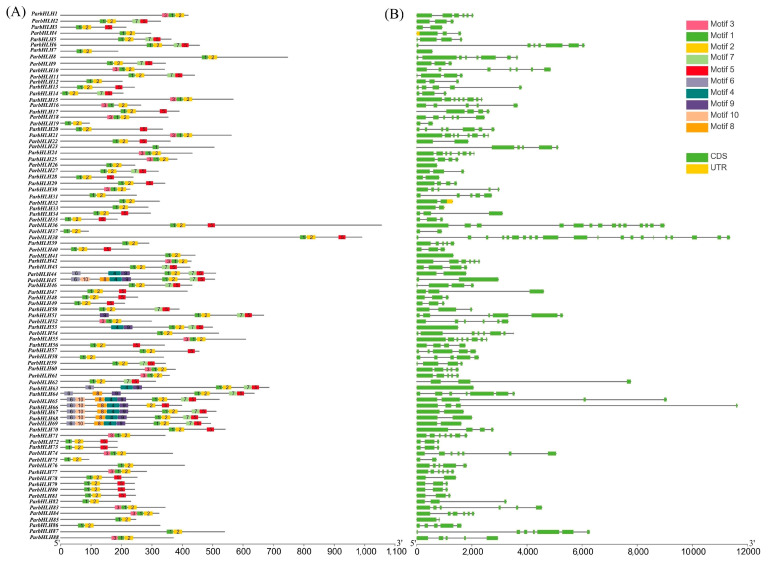
Gene structure and conserved motif analysis of the *bHLH* genes. (**A**) Structure of *ParbHLH* genes. (**B**) Distribution of 10 conserved motifs of bHLH proteins.

The gene structure and conserved motifs of ParbHLHs were analyzed in order to understand the structure and function of the *bHLH* gene family in apricot. As shown in Figure 6A, the gene structure and conserved motifs of ParbHLHs were analyzed by MEME, which showed that a total of 10 conserved protein motifs were obtained. All of these sequences exhibited two types of highly conserved protein motifs, respectively, shown as green (motif 1) and yellow (motif 2) blocks. These two conserved domains were shown to be adjacent to each other, and the gap between motif 1 and motif 2 varied depending on the loop length. As shown in Figure 6B, it is indicated by green and yellow boxes for CDS and UTR, respectively, and black lines for introns in the intron and exon maps. The most highly conserved *ParbHLH* genes were found to share a common structure, with the number of introns ranging from 0 (five genes with no introns) to 20 (*ParbHLH38*). Conservative motif and gene structure analyses suggested that *ParbHLH65*, *ParbHLH66*, *ParbHLH67*, *ParbHLH68*, and *ParbHLH69* may have similar biological functions.

#### 2.6.5. Chromosomal Location and Gene Co-Linearity Analysis of bHLH Family Genes in Apricot

**Figure 7:**
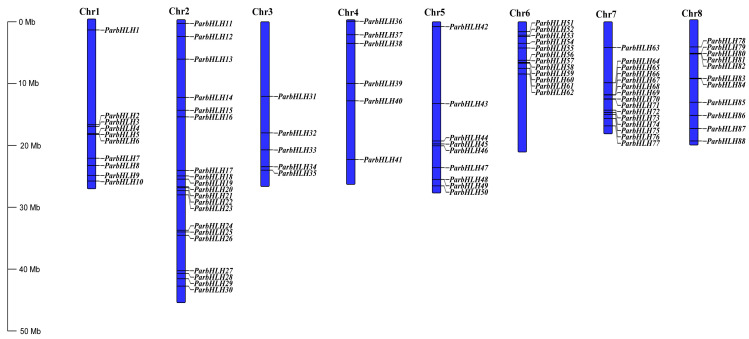
Gene localization and distribution of *bHLH* genes on chromosomes.

As shown in Figure 7, the apricot genome contained eight chromosomes (Chr1-Chr8) (2n = 16), with the highest number of *ParbHLH* genes (20) on chromosome 2, followed by chromosome 7 (15), chromosome 6 (12), chromosome 8 (11), chromosome 1 (10), chromosome 5 (9), chromosome 4 (6) and chromosome 3 (5). To investigate whether tandem repeats contribute to the amplification of the *bHLH* gene during evolution, the non-synonymous/synonymous mutation (Ka/Ks) ratio of the gene was calculated. The results indicated that the 17 *ParbHLH* genes were composed of nine gene pairs, with tandem duplicated genes accounting for 19.32% of the whole gene family (Table S13). The genes were replicated on eight chromosomes, with the highest number of genes on chromosome 7 (7), followed by chromosome 6 (4) and chromosomes 1, 5, and 8 (respectively, two genes). Gene clusters were observed on chromosome 7 (*ParbHLH65*, *ParbHLH66* and *ParbHLH67*, *ParbHLH68* and *ParbHLH69*). The Ka/Ks ratio can be used as an indicator of genetic selection pressure during evolution. The ratios of duplicated *bHLH* gene pairs (except *ParbHLH72*/*ParbHLH73*) in ‘Xiaobaixing’ were <1, indicating that genes evolved were mainly under the influence of purifying selection. Co-linearity analysis of the *Arabidopsis* and ‘Xiaobaixing’ genomes showed that the gene clusters were co-linear with multiple *bHLH* genes in *Arabidopsis* (Figure S2).

#### 2.6.6. Analysis of Cis-Regulatory Elements of the *bHLH* Gene Family Promoter

**Figure 8:**
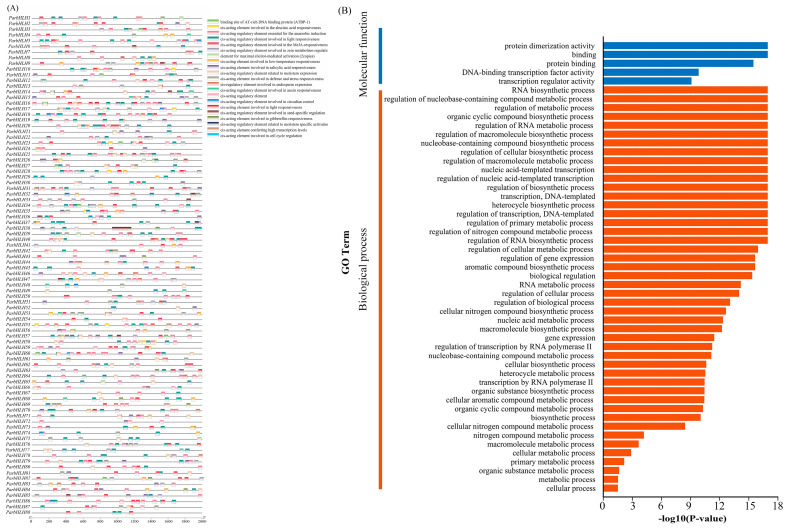
Identification of key cis-elements in the promoter of the *bHLH* genes and GO annotation analysis. (**A**) Identification of key cis-elements. (**B**) GO annotation analysis.

The promoters of genes contained cis-regulatory elements that could potentially reveal gene function [34,35]. To investigate the pattern of regulation of gene expression, cis-regulatory elements 2000 bp upstream of the transcription start site (promoter region) of *ParbHLH* family genes were analyzed using PlantCARE. As shown in Figure 8A, 21 functionally annotated cis-regulatory elements were identified in the promoters of 88 *ParbHLH* genes, which mainly included regulatory elements such as light responsiveness elements, defense and stress responsiveness elements, hormone response elements, and genes involved in metabolite biosynthesis. In addition, regulatory elements involved in seed-specific regulation (RY-element) and endosperm expression (AACA_motif) were also identified. Cis-acting elements involved in meristem expression, anaerobic induction, salicylate response, abscisic acid response, gibberellin response, low-temperature response, and light response were mainly included in the promoters of *ParbHLH66* genes.

#### 2.6.7. GO Annotation and Protein Interactions of the *bHLH* Family Genes of Apricot

As shown in Figure 8B, GO annotation was performed for all *ParbHLH*, and 88 *ParbHLH* were annotated into two functional categories: biological process (BP) and molecular function (MF), respectively. In the category of biological processes, *ParbHLH* genes were involved in the regulation of RNA biosynthetic processes (n = 26), nucleobase-containing compound biosynthetic processes (n = 26), the regulation of macromolecule metabolic processes (n = 25), the regulation of primary metabolic processes (n = 25), RNA biosynthetic processes (n = 25), the regulation of cellular metabolic processes (n = 25), regulation of gene expression (n = 25), organic substance biosynthetic processes (n = 27), metabolic processes (n = 28), and cellular processes (n = 30) were significantly enriched. In the molecular function category, genes were enriched in protein dimerization activity (n = 88), binding (n = 88), protein binding (n = 88), DNA-binding transcription factor activity (n = 19), and transcriptional regulator activity (n = 19). In conclusion, the functions of the *ParbHLH* gene were mainly involved in metabolic processes, nucleotide-binding TF activity, and catalytic activity in bioregulation, cells, developmental processes, and metabolism of single organisms.

#### 2.6.8. Analysis of the Prediction of Protein Interaction Networks for ParbHLH

**Figure 9:**
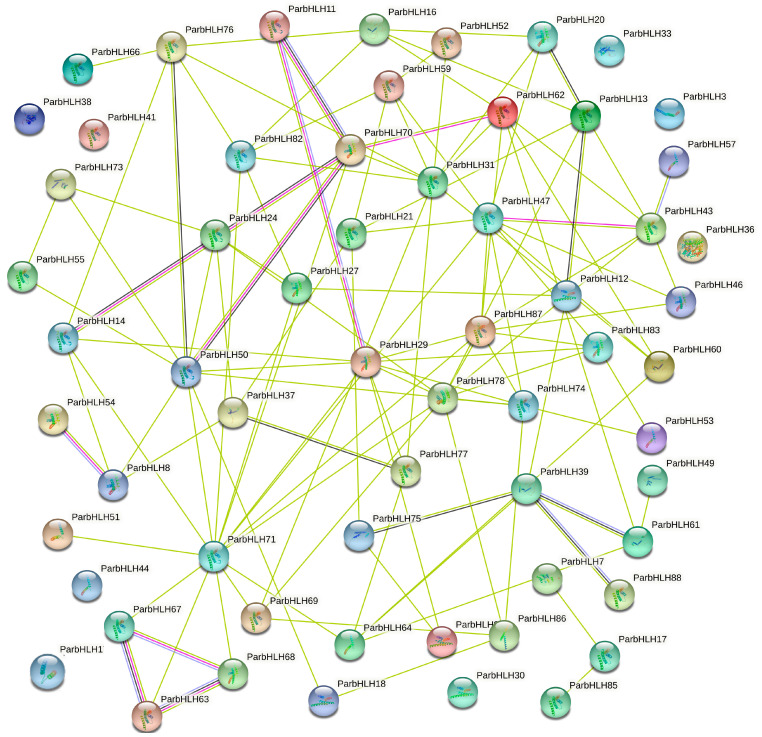
ParbHLH protein interaction network based on *Arabidopsis thaliana*.

To further understand the regulatory network of *bHLH* transcription factors, the protein–protein interaction network of ParbHLH with other proteins in the *Arabidopsis* genome was constructed by STRING software analysis. As shown in Figure 9, a total of 80 ParbHLH proteins were predicted to have protein-interaction relationships, and eight ParbHLH (ParbHLH1, ParbHLH3, ParbHLH30, ParbHLH33, ParbHLH36, ParbHLH38, ParbHLH41, and ParbHLH44) did not interact with other family members. On the other hand, more than 30 proteins interact with at least four other ParbHLH proteins, with ParbHLH71 having the most interactions. The 30 ParbHLH proteins, especially ParbHLH71, were thought to play an important role in plant growth and development. Overall, the results provided an important reference for identifying the true interactions of ParbHLH with biochemical experiments.

## 3. Discussion

### 3.1. Genome Evolution

Amygdalin is considered an important indicator for the selection and breeding of bitter apricot seeds. The sweetness/bitterness of its seed is mainly determined by the amygdalin content, and the regulation of the accumulation of bitter substances is a key issue in improving the quality of apricot seed. To clarify amygdalin biosynthesis from the genetic level, the genome of ‘Xiaobaixing’ was sequenced in this study by PacBio Sequel II + BGI MGISEQ-2000 PE150 sequencing, Hi-C assisted and BUSCO evaluation combined technology assembly, with final anchored to chromosome length of 0.21 Gb, with 52.80% repeat sequences as a proportion of the genome, 29,157 protein-coding genes predicted, contigs N50 = 3.56 Mb, scaffold N50 = 26.73 Mb, contig length anchoring rate of 92.23%, and contig number anchoring rate of 32.10%. This is compared with the published genome of the ‘String Red’ apricot constructed based on genetic mapping [30], whose eight constructed pseudomolecules were lower in size and N50 than the genome constructed in this study. Compared with the ‘Meihua’ apricot genome assembled by Yuan Kejun [31], the ‘Xiaobaixing’ apricot genome had a lower mount rate than ‘Meihua’, but contigs N50 and the number of predicted genes was higher than that of ‘Meihua’. Compared with the ‘Yinxiangbai’ apricot genome [32], its repeat sequence was lower than that of the ‘Xiaobaixing’ genome. Various germplasm had an effect on the size of the assembled genome, the size of the N50, the size of the pseudomolecules, and the number of predicted genes. For example, gene number predictions for apples yielded different numbers of genes, 63,141, 53,922, and 42,140, respectively [36,37,38]. The above study indicates that the ‘Xiaobaixing’ genome is assembled with high quality in this study, which can provide a reference for conducting related gene studies and subsequent breeding studies to investigate important traits.

A comparison of genomes showed that ‘Yinxiangbai’ and ‘Xiaobaixing’ diverged by 5.3 Ma, with apricot being most closely related to *P. mume* [39], and both diverged by 9.6 Ma. The common ancestor of *P. mume* diverged from *P. armeniaca* and *P. salicina* by 12.2 Ma, and the common ancestor of *P. persica* diverged from *P. mume*, *P. armeniaca*, and *P. salicina* by 14.0 Ma, and the ancestor of *P. avium* diverged even earlier, by about 17.8 Ma. The result was consistent with the phylogenetic relationships studied, but there was some variation in the time of divergence, which may be related to the different comparison plants used [31]. The results of Yuan Kejun et al. [31] showed that *P. persica* and *P. dulcis* are closely related, but the number of single-copy genes was less than 100 with the addition of *P. dulcis*, so *P. dulcis* was not added for phylogeny in this study, and since the only genome assembled to chromosomes with complete annotation information on NCBI was that of ‘Yinxiangbai’ when the comparative genomes were done, only this genome was compared in this study. In summary, the evolutionary developmental history of the Rosaceae has been analyzed in this study, while the whole genomes of different apricot germplasm remain to be studied.

Traits are determined by genes, and the expansion and contraction of gene families play a key role in the phenotypic differentiation of plants [40]. Based on a phylogenetic tree constructed for 15 plant species, 569 expansion genes, and 1316 contraction genes were found for ‘Xiaobaixing’, with 227 gene families unique to the species. Genomic analysis revealed a significant expansion of the phenylalanine *N-monooxygenase* and β-glucosidase genes. Significant contraction occurred in the wild black cherry glucoside β-glucosidase gene, amygdalin β-glucosidase gene, and β-glucosidase gene. Positive selection genes were significantly enriched in the cyanogenic amino acid metabolic pathway. The above results suggested that these genes may be associated with the biosynthesis of amygdalin in apricot. In addition, ‘Xiaobaixing’ has low carotenoid content, which may be due to a significant contraction of genes related to the synthesis of carotenoids. The sweet flavor and high unsaturated fatty acid content of ‘Xiaobaixing’ kernels may be due to a significant expansion of genes related to the synthesis of various substances such as α-linolenic acid, linoleic acid, starch, and sucrose, which deserves further study.

### 3.2. bHLH Transcription Factor Allowed Apricot Domestication

Numerous studies have shown that the biosynthesis of bitter amygdalin depends on phenylalanine metabolism and that the *bHLH* gene family is involved in the synthesis of bitterness [23]. A total of 88 *ParbHLH* genes were identified, and these were distributed in eight chromosomes. The amino acids were mainly composed of Ser, Glu, Arg, and Leu, which were necessary for binding to DNA sequences [41]. The expansion of gene families during evolution was mainly the result of repetitive events [42]. The presence of segmental and tandem replication of the *ParbHLH* gene in this study suggests that gene replication events may play an important role in the amplification of the *bHLH* gene in ‘Xiaobaixing’. Analysis of the cis-acting elements of the *ParbHLH* gene family has reveal that the promoter of the gene family contains three main elements, namely light, stress, and hormone, and it is speculated that the gene family plays an important role in growth and development and stress resistance. Furthermore, the presence of a bHLH binding site (G-box) and the specific expression of the endosperm suggested that the *ParbHLH* transcription factors could regulate each other or interact with others in the growth and development of ‘Xiaobaixing’, which was consistent with the GO annotation and the predicted results of the PPI network. The results of chromosomal localization and non-synonymous/synonymous mutation (Ka/Ks) ratios of genes indicated that *ParbHLH66* (*rna-Par24659.1*) were homologous to *ParbHLH65*, *ParbHLH67*, *ParbHLH68*, and *ParbHLH69* genes and clustered into one gene cluster. Cluster analysis showed that *ParbHLH66* (*rna-Par24659.1*) was highly homologous to the *bHLH2* gene, which regulates sweet/bitter traits in *P. dulcis*, and hypothesized that the gene cluster might be involved in the biosynthesis of amygdalin in apricot kernels.

In this study, the key pathway and regulatory genes of amygdalin in apricot kernels were excavated by genome sequencing technology, and the *bHLH* gene family involved in bitterness regulation was identified based on bioinformatics technology. A candidate gene closely associated with the regulation of the kernels was identified as *ParbHLH66* (*rna-Par24659.1*). However, it remains to be further investigated how this gene is specifically involved in regulation and, if not, the other transcription factors bind to the *bHLH* gene for the regulation of amygdalin. This study provided a theoretical basis for revealing the regulation and biosynthesis of sweet/bitter apricot kernels, laying the foundation for breeding kernel types with high yield and low amygdalin, and providing new insight into the potential mechanisms of adaptive domestication of apricot.

## 4. Materials and Methods

### 4.1. Plant Material and Genome Sequencing

Fresh and young leaves of ‘Xiaobaixing’ from the germplasm resource of Tarim University (Alar, China) were selected as the study material, which was washed and treated with 75% ethanol and sterile water, quickly frozen and ground in liquid nitrogen, stored at −80 °C, and DNA was obtained using the DNA extraction kit (Tiangen, Beijing, China) and the libraries were purified, end-repaired, A-added, ligated, and PCR-amplified. The quality-checked libraries were sequenced using the MGIseq 2000 platform based on effective concentrations and target downstream data volumes. Genome sequencing was performed by Frasergen (Wuhan, China). Genomic data were submitted to the BIG Submission (https://bigd.big.ac.cn/gsub/ (accessed on 12 June 2023)) with access number PRJCA017645.

### 4.2. Genome Assembly and Annotation

The clean data was obtained by QC from the raw data, followed by the data that were compared and filtered using Juicer software, and the sequencing quality and error rate were normal. Prodigal software was used to perform ORF (open reading frame) prediction on the assembled contig sequences, and CD-HIT software was used to de-redundancy the predicted results to obtain a non-redundant gene set. The sequencing data were compared to the constructed non-redundant gene sets using Bowtie software, and information on the abundance of individual genes in different samples was counted. The predicted non-redundant gene sets were compared and annotated with the functional annotation databases InterPro, GO, KEGG ALL, KEGG KO, SwissProt, TrEMBL, and NR.

### 4.3. Analysis of Phylogeny and Estimation of Divergence Times

The MEGA7.0 software was used to construct molecular evolutionary trees of annotated genes from 15 plants, including apricot, *P. mume*, and *P. persica*, and analyze their affinities with the Rosaceae genus (Table S14). Time correction points were obtained in combination with TimeTree, and divergence times were estimated by r8s and PAML software.

### 4.4. Expansion and Contraction Analysis, Positive Selection Analysis and Covariance Analysis on Gene Families

Gene family expansion and contraction analysis were performed by CAFE software. GO/KEGG enrichment analysis was performed for members of the significantly expanded and contracted gene family. Both the GO terms and KEGG terms with *q*-value ≤ 0.05 were considered to be significantly enriched. Based on the gene family clustering results for each shared single-copy direct homologous gene family, the gene was tested for positive selection by the branch-site model using codeml in PAML. For genes that were positively selected, GO/KEGG enrichment analysis was then performed. The assembled genomic sequences were aligned with the reference genomic sequences using Mummer, and a covariance scatter plot was obtained based on jcvi mapping.

### 4.5. Analysis of the bHLH Gene Family in Apricot

#### 4.5.1. Identification of the bHLH Gene

The *bHLH* family protein sequences of *Arabidopsis* were downloaded to build the *Arabidopsis* Information Resource Database (http://www.arabidopsis.org/ (accessed on 6 May 2021)). Download the Hidden Markov Model (HMM) file (PF00010) for the *bHLH* structural domain from the Pfam database (http://pfam.xfam.org/ (accessed on 6 May 2021)), use HMMER for protein filtering (E value of 1 × 10^−5^), set the parameter --cut_ga, use TBtools to extract the target sequences and search in BLASTP. The screening was performed using the CDD conserved domain database in NCBI and the simple modular architecture research tool known as SMART, which ultimately yielded the apricot *bHLH* transcription factor.

#### 4.5.2. Bioinformatics Analysis of the *bHLH* Gene Family

Analysis of the nucleotide profile of the *bHLH* gene family in apricot using SMS analysis software (http://www.bioinformatics.org/sms2/dna_stats.htm1 (accessed on 6 May 2021)). Physicochemical characterization of the *bHLH* gene family by ProtParain software and gene structure analysis of *bHLH* by TBtools. Determination of the non-synonymous/synonymous mutation (Ka/Ks) ratio of genes based on chromosomal location and gene covariance analysis of the identified *bHLH* family genes. Analysis of conserved cis-regulatory elements in the promoter region of the *bHLH* gene by the PlantCARE online tool; protein interaction network by STRING. Prediction of motifs in the bHLH protein of apricot by MEME online software (http://meme-suite.org/tools/meme (accessed on 6 May 2021)); construction of phylogenetic trees by MAGA7.0, calculated by Bootstrap with 1000 replicates.

## 5. Conclusions

Here, we report the high-quality chromosomal level genome assembly of ‘Xiaobaixing’, meanwhile 88 *bHLH* genes were identified, and *ParbHLH66* (*rna-Par24659.1*) was found to be a key gene for sweet/bitter kernels. These genes may be involved in the regulation of amygdalin biosynthesis, providing new insights into the potential mechanisms of adaptive domestication.

## Data Availability

Data are available in a publicly accessible repository. The refined genome data presented in this study are openly available at the BIG Submission (NGDC). Accession number: PRJCA017645.

## Supplementary Materials

The following supporting information can be downloaded at: https://www.mdpi.com/article/10.3390/plants12152756/s1, Figure S1: Topography of the apricot genome, Figure S2: Gene covariance analysis of the *bHLH* genes in apricot, Table S1: Sequencing statistics of the genome from ‘Xiaobaixing’, Table S2: Genomic signature statistics (K-mer = 17), Table S3: Statistics data, Table S4: Comparison statistics, Table S5: Comparison statistics, Table S6: Hi-C assisted assembly overall statistics of apricot, Table S7: Genomic statistics results of different prediction methods, Table S8: Statistics of non-coding RNA annotation results, Table S9: Clustering statistics, Table S10: Statistical results of significantly expanded and contracted genes in ‘Xiaobaixing’, Table S11: A comparison nucleic acid sequences of *bHLH* genes, Table S12: A comparison of composition, physical and chemical characters of *bHLH* genes, Table S13: Estimated Ka/Ks ratios of the duplicated *bHLH* gene in apricot, Table S14: Addresses of 15 species genomes.
