# Supplementary material for: A Chromosome-Level Genome of ‘Xiaobaixing’ (Prunus armeniaca L.) Provides Clues to Its Domestication and Identification of Key bHLH Genes in Amygdalin Biosynthesis
Source: Plants (Basel). 2023 Jul 25;12(15):2756. doi: 10.3390/plants12152756 (PMC10421183; doi:10.3390/plants12152756)
Supplement: Supplementary file 1 [file plants-12-02756-s001.zip › Supplementary Table.pdf]

Table S1 Sequencing statistics of the genome from 'Xiaobaixing'.

data type	Read Num	Base Count /bp	Read Length /bp	Q20 /%	Q30 /%	GC Content %
raw data	88106970x2	13216045500;13216045500	150;150	97.0;96.4	89.3;88.4	42.7;42.8
clean data	78830883x2	11824632450;11824632450	150;150	97.0;96.6	89.3;88.7	42.0;42.1

Table S2 Genomic signature statistics（K-mer=17）

K-mer number	K-mer Depth	Genome Size（Mb）	Heterozygous Ratio /%	Repeat /%
19298819628	39	451.72	0.98	68.94

Table S3 Statistics data

Name	scaffold length（bp）	scaffold number	contig length（bp）	contig number
Max len	45 787 477	-	18 885 238	-
N10	45 787 477	1	14 302 944	2
N20	27 707 315	2	9 754 762	4
N30	27 707 315	2	6 008 335	7
N40	27 475 951	3	4 294 639	11
N50	26 685 924	4	3 183 073	18
N60	26 665 821	5	2 421 308	26
N70	21 092 742	6	1 381 177	39
N80	20 299 355	7	974 010	58
N90	18 149 216	8	375 464	95
Total length	231 886 072	302	231 826 282	431

Note: max len: the length of the longest scaffold and contig; N50: the length of scaffold and contig bases with length greater than or equal to N50 and their numbers, and so on; Total length: the total length of all scaffold and contig bases and their numbers.

Table S4 Comparison statistics

data type	mapping rate /%	average sequencing depth	coverage /%	Coverage (>= 5X,%)	Coverage (>= 10X,%)	Coverage (>= 20X,%)
Second	96.18	99.82	99.23	98.75	98.04	94.11
Third	99.95	78.77	99.7	98.9	97.99	94.58

Note: Mapping rate: reading rate of contig on reads comparison; Average sequencing depth: average sequencing depth (= single base depth sum/total number of bases); Coverage: coverage (= the number of bases with depth not less than 1/total number of bases); Coverage (≥5X): 5X coverage (= the number of bases with depth not less than 5/total number of bases); Coverage (≥10X): 10X coverage (= the number of bases with depth not less than 10/total number of bases); Coverage (≥20X): 20X coverage (= the number of bases with depth not less than 20/total number of bases).

Table S5 Comparison statistics

Term	Genes	Percentage (%)
Complete BUSCOs	1 552	96.2
Complete and single-copy BUSCOs	1 514	93.8
Complete and duplicate BUSCOs	38	2.4
Fragmented BUSCOs	6	0.4
Missing BUSCOs	56	3.5
Total BUSCO groups searched	1614	100

Note: Complete BUSCOs: genes with complete BUSCO profiles; Complete and single-copy BUSCOs: a BUSCO with a single gene; Complete and duplicated BUSCOs: a BUSCO with multiple genes. Fragmented BUSCOs: genes with only partial sequences that match the BUSCO profile; Missing BUSCOs: genes that do not match the BUSCO profile; Total BUSCO groups searched: total number of genes in BUSCO groups.

Table S6 Hi-C assisted assembly overall statistics of apricot

	序列总长度 Total /bp	Contig num	Contigs N50 /bp	Scaffold num	Scaffold N50 /bp	GC Content /%	Contig Number (>100kb)	Contig Length (>100kb)	Contig Length Proportion (>100kb)
Original sequence	232205771	433	3185241	-	-	42.7; 42.8	142	219117986	-
Chromosome sequence	214168809	139	3559988	8	26727503	42.0; 42.1	124	213517693	97.44%

Note: Total: Total sequence length, excluding Gap; Contig Num: Contig number, if there is an assembly error inside Contig, Contig will be cut off from the error; Contig N50: Contig is cut off due to the presence of error inside Contig, so Contig N50 decreases; Scaffold Num: the number of Scaffold, i.e. the number of chromosomes; Contig Number(>100kb): the number of Contig entries greater than 100kb. Contig Length(>100kb): the total length of Contig greater than 100kb.

Table S7 Genomic statistics results of different prediction methods

Gene set	Number	Average gene length /bp	Average CDS length /bp	Average exon per gene	Average exon length /bp	Average intron length /bp
Augustus	17 135	3 684.07	1 546.69	6.12	252.88	417.77
GlimmHMM	23 605	7 589.46	1 067.80	4.57	233.86	1 828.87
Genscan	16 950	8 103.61	1 446.40	6.81	212.41	1 145.92
GeneID	33 693	3 546.01	841.59	4.29	196.34	822.94
杏（串枝红）基因序列同源预测	46 979	1 963.20	753.67	3.00	250.85	603.41
杏（银香白）基因序列同源预测	44 144	2 287.36	799.42	3.18	251.73	683.90
梅 Prunus mume Siebold & Zucc.基因序列同源预测	35 510	2 645.44	995.18	3.69	269.96	614.30
李（Prunus salicina Lindl.）基因序列同源预测	45 882	2 267.65	855.04	3.24	264.04	631.12
桃(Prunus persica (L.) Batsch）基因序列同源预测	35 186	2 498.31	897.01	3.58	250.54	620.60
MAKER 整合	29 368	2 878.72	1 029.69	4.27	242.65	564.46

PASA 更新	29 157	2 886.86	1 036.09	4.29	243.01	563.53

Table S8 Statistics of non-coding RNA annotation results

ncRNA type	Segment type	Copy	Average length /bp	Total length /bp	Genome /%
miRNA		153	113.67	17 392	0.01
tRNA		641	75.88	48 640	0.02
rRNA	rRNA	2 671	3 465.53	9 256 441	3.99
	18S	1 215	1 799.02	2 185 809	0.94
	28S	1 212	5 810.71	7 042 583	3.04
	5S	244	114.95	28 049	0.01
snRNA	snRNA	401	114.52	45 922	0.02
	CD-box	257	100.91	25 935	0.01
	HACA-box	38	130.32	4 952	0.00
	splicing	106	141.84	15 035	0.01
	scaRNA	0	0.00	0	0.00

Note: miRNA: MicroRNA, degrades mRNA or inhibits its translation, and has the function of silencing genes; tRNA: transfer RNA, carries amino acids into ribosomes to synthesize proteins under the guidance of mRNA; rRNA: ribosomal RNA, binds to proteins to form ribosomes, and its function is to act as a scaffold for mRNA, providing a mRNA translation into protein 18S, 28S, 5S, all belong to rRNA, S is a physical unit of macromolecules in ultracentrifugal sedimentation, which can indirectly reflect the size of molecular weight; snRNA: small nuclear RNA, mainly involved in the processing of RNA precursors, is the main component of RNA shear body. cd-box, HACA-box, splicing, scaRNA all belong to snRNA.

Table S9 Clustering statistics

Species	Genes number	Family number	Unique families	Single copy	Average genes per family
P. armeniaca L. (‘Xiaobaixing’)	29 157	18 311	227	149	1.48
P. armeniaca (‘yinxiangbai’)	28 677	17 820	174	149	1.51

P. mume	22 708	15 541	9	149	1.46
P. persica	24 084	15 983	145	149	1.46
P. avium	24 367	15 620	30	149	1.55
P. salicina	29 714	17 162	95	149	1.70
M. domestica	41 920	17 320	825	149	2.22
P. bretschneideri	33 957	14 746	123	149	2.25
E. japonica	41 297	16 278	346	149	2.42
G. trifoliata	24 675	14 707	388	149	1.62
R. rugosa	35 110	15 685	536	149	2.17
R. occidentalis	28 766	16 206	812	149	1.55
F. vesca	23 724	15 739	79	149	1.49
P. micrantha	33 602	16 604	876	149	1.79
A. chinensis	31 609	14 601	610	149	2.08
Z. jujuba	28 450	15 031	585	149	1.81

Table S10 Statistical results of significantly expanded and contracted genes in 'Xiaobaixing'

	KEGG Pathway	Pathway ID	Q-Value	Gene IDs
Contraction gene	Cyanoamino acid metabolism	ko00460	3.60E-10	rna-Par06928.1,rna-Par21289.1,rna-Par00213.1,rna-Par06929.1, rna-Par01447.1,rna-Par01109.1,rna-Par26932.1,rna-Par26931.1, rna-Par11192.1
	Fatty acid elongation	ko00062	6.85E-10	rna-Par09809.1,rna-Par07916.1,rna-Par03992.1,rna-Par19695.1, rna-Par21936.1,rna-Par08831.1,rna-Par07208.1
	Phenylpropanoid biosynthesis	ko00940	1.16E-09	rna-Par21608.1,rna-Par21612.1,rna-Par01447.1,rna-Par21610.1, rna-Par06929.1,rna-Par21289.1,rna-Par25243.1,rna-Par06928.1, rna-Par01672.1,rna-Par14360.1,rna-Par26932.1,rna-Par11192.1, rna-Par21609.1

	Stilbenoid, diarylheptanoid and gingerol biosynthesis	ko00945	5.16E-02	rna-Par25243.1
	Carotenoid biosynthesis	ko00906	5.16E-02	rna-Par21614.1
	Amino sugar and nucleotide sugar metabolism	ko00520	7.06E-02	rna-Par04593.1,rna-Par21210.1
	Flavonoid biosynthesis	ko00941	7.96E-02	rna-Par25243.1
	Plant hormone signal transduction	ko04075	3.14E-01	rna-Par18479.1,rna-Par24317.1
Expansion gene	Indole alkaloid biosynthesis	ko00901	0.00E+00	rna-Par14011.1,rna-Par12298.1,rna-Par20772.1,rna-Par02175.1, rna-Par12302.1,rna-Par12297.1,rna-Par12305.1
	Pentose and glucuronate interconversions	ko00040	7.96E-17	rna-Par09768.1,rna-Par08856.1,rna-Par14260.1,rna-Par09770.1, rna-Par18115.1,rna-Par25744.1,rna-Par16662.1,rna-Par16646.1, rna-Par14263.1,rna-Par22212.1,rna-Par14269.1,rna-Par14272.1, rna-Par16647.1,rna-Par08863.1,rna-Par14271.1,rna-Par16648.1, rna-Par18114.1,rna-Par16659.1,rna-Par14267.1,rna-Par14274.1, rna-Par14270.1,rna-Par14275.1,rna-Par14261.1,rna-Par22213.1, rna-Par16658.1,rna-Par16649.1,rna-Par16660.1,rna-Par16663.1
	Cyanoamino acid metabolism	ko00460	5.64E-14	rna-Par27165.1,rna-Par01567.1,rna-Par01527.1,rna-Par01529.1, rna-Par27166.1,rna-Par01564.1,rna-Par00648.1,rna-Par01532.1, rna-Par27175.1,rna-Par27172.1,rna-Par17933.1,rna-Par26024.1, rna-Par27168.1,rna-Par01562.1,rna-Par27173.1,rna-Par01526.1, rna-Par27178.1,rna-Par17344.1
	Betalain biosynthesis	ko00965	7.79E-12	rna-Par02175.1,rna-Par20772.1,rna-Par12302.1,rna-Par12305.1, rna-Par12297.1,rna-Par12298.1,rna-Par14011.1
	Plant hormone signal transduction	ko04075	1.90E-10	rna-Par22116.1,rna-Par22111.1,rna-Par22107.1,rna-Par22112.1, rna-Par21407.1,rna-Par22108.1,rna-Par22101.1,rna-Par04682.1, rna-Par21402.1,rna-Par04686.1,rna-Par22118.1,rna-Par22099.1,

			rna-Par22102.1,rna-Par22117.1,rna-Par11972.1,rna-Par22104.1, rna-Par21403.1,rna-Par22096.1,rna-Par11973.1,rna-Par21399.1, rna-Par21362.1,rna-Par22109.1,rna-Par04680.1,rna-Par22113.1, rna-Par21855.1,rna-Par21405.1,rna-Par21367.1,rna-Par04481.1, rna-Par22097.1
alpha-Linolenic acid metabolism	ko00592	2.72E-09	rna-Par12715.1,rna-Par20979.1,rna-Par06329.1,rna-Par20977.1, rna-Par12716.1,rna-Par20974.1,rna-Par20973.1,rna-Par20981.1, rna-Par12718.1,rna-Par20975.1,rna-Par16125.1,rna-Par20971.1, rna-Par20976.1
Glucosinolate biosynthesis	ko00966	1.51E-08	rna-Par27166.1,rna-Par27175.1,rna-Par27172.1,rna-Par27178.1, rna-Par27165.1,rna-Par27168.1,rna-Par27173.1
Lysine biosynthesis	ko00300	1.35E-07	rna-Par09191.1,rna-Par09201.1,rna-Par09701.1,rna-Par21385.1, rna-Par09202.1,rna-Par09197.1,rna-Par09195.1
Phenylpropanoid biosynthesis	ko00940	1.35E-07	rna-Par01567.1,rna-Par09677.1,rna-Par09679.1,rna-Par01527.1, rna-Par01529.1,rna-Par00481.1,rna-Par00479.1,rna-Par00648.1, rna-Par01564.1,rna-Par00482.1,rna-Par01532.1,rna-Par17933.1, rna-Par26024.1,rna-Par00478.1,rna-Par00483.1,rna-Par00480.1, rna-Par01562.1,rna-Par01526.1,rna-Par09676.1,rna-Par09680.1, rna-Par17344.1
Phenylalanine metabolism	ko00360	1.68E-05	rna-Par20772.1,rna-Par02175.1,rna-Par12302.1,rna-Par12297.1, rna-Par12305.1,rna-Par14011.1,rna-Par12298.1
Linoleic acid metabolism	ko00591	1.85E-05	rna-Par12718.1,rna-Par12716.1,rna-Par16125.1,rna-Par12715.1, rna-Par06329.1
Isoquinoline alkaloid biosynthesis	ko00950	3.63E-05	rna-Par14011.1,rna-Par12298.1,rna-Par12305.1,rna-Par12302.1, rna-Par12297.1,rna-Par20772.1,rna-Par02175.1
Metabolism of xenobiotics by	ko00980	7.25E-04	rna-Par18050.1,rna-Par29117.1,rna-Par29032.1,rna-Par29035.1,

cytochrome P450			rna-Par18049.1,rna-Par18047.1,rna-Par29114.1
Drug metabolism - cytochrome P450	ko00982	1.05E-03	rna-Par29114.1,rna-Par18047.1,rna-Par18049.1,rna-Par29032.1, rna-Par29035.1,rna-Par29117.1,rna-Par18050.1
Sesquiterpenoid and triterpenoid biosynthesis	ko00909	1.51E-03	rna-Par16265.1,rna-Par26304.1,rna-Par03763.1,rna-Par16260.1
Starch and sucrose metabolism	ko00500	2.51E-03	rna-Par00648.1,rna-Par01526.1,rna-Par01564.1,rna-Par01532.1, rna-Par17933.1,rna-Par26024.1,rna-Par17344.1,rna-Par01567.1, rna-Par01562.1,rna-Par01527.1,rna-Par01529.1
Drug metabolism - other enzymes	ko00983	3.83E-03	rna-Par29117.1,rna-Par18050.1,rna-Par29032.1,rna-Par29035.1, rna-Par18049.1,rna-Par18047.1,rna-Par29114.1
Quorum sensing	ko02024	1.33E-02	rna-Par08863.1,rna-Par25744.1,rna-Par08856.1,rna-Par09770.1, rna-Par09768.1
Steroid biosynthesis	ko00100	1.33E-02	rna-Par04417.1,rna-Par04416.1,rna-Par16266.1
Glutathione metabolism	ko00480	1.46E-02	rna-Par29117.1,rna-Par18050.1,rna-Par29035.1,rna-Par29032.1, rna-Par18049.1,rna-Par18047.1,rna-Par29114.1
Ascorbate and aldarate metabolism	ko00053	2.70E-02	rna-Par00013.1,rna-Par00012.1,rna-Par00011.1,rna-Par00014.1

Table S11 A comparison of nucleic acid sequences of *bHLH* genes

Gene name	Gene ID	length /bp	Number of bases A	Number of bases C	Number of bases G	Number of bases T	G+C content/%	A+T content /%
ParbHLH1	*rna-Par00237.1*	1260	385	262	287	326	43.57	56.43
ParbHLH2	*rna-Par02090.1*	987	288	208	251	240	46.5	53.5
ParbHLH3	*rna-Par02133.1*	648	189	115	202	142	48.92	51.08
ParbHLH4	*rna-Par02242.1*	891	284	176	205	226	42.76	57.24

ParbHLH5	*rna-Par02260.1*	1092	331	248	227	286	43.5	56.5
ParbHLH6	*rna-Par02261.1*	1371	406	327	272	366	43.69	56.31
ParbHLH7	*rna-Par02896.1*	567	159	144	118	146	46.21	53.79
ParbHLH8	*rna-Par03127.1*	2241	657	446	545	593	44.22	55.78
ParbHLH9	*rna-Par03394.1*	1035	296	248	227	264	45.89	54.11
ParbHLH10	*rna-Par03539.1*	1026	263	228	295	240	50.97	49.03
ParbHLH11	*rna-Par03812.1*	1323	399	282	342	300	47.17	52.83
ParbHLH12	*rna-Par04057.1*	609	190	115	177	127	47.95	52.05
ParbHLH13	*rna-Par04530.1*	729	250	129	178	172	42.11	57.89
ParbHLH14	*rna-Par05320.1*	618	191	124	138	165	42.39	57.61
ParbHLH15	*rna-Par05524.1*	1704	509	368	407	420	45.48	54.52
ParbHLH16	*rna-Par05639.1*	792	237	159	198	198	45.08	54.92
ParbHLH17	*rna-Par06576.1*	1170	366	228	274	302	42.91	57.09
ParbHLH18	*rna-Par06710.1*	1062	285	232	304	241	50.47	49.53
ParbHLH19	*rna-Par06794.1*	285	77	71	71	66	49.82	50.18
ParbHLH20	*rna-Par07020.1*	1008	309	223	245	231	46.43	53.57
ParbHLH21	*rna-Par07060.1*	1686	470	350	448	418	47.33	52.67
ParbHLH22	*rna-Par07140.1*	1083	323	291	248	221	49.77	50.23
ParbHLH23	*rna-Par07267.1*	1515	435	394	300	386	45.81	54.19
ParbHLH24	*rna-Par08322.1*	1299	386	257	311	345	43.73	56.27
ParbHLH25	*rna-Par08385.1*	1149	361	223	264	301	42.38	57.62
ParbHLH26	*rna-Par08476.1*	735	216	215	149	155	49.52	50.48
ParbHLH27	*rna-Par09353.1*	966	295	222	228	221	46.58	53.42

ParbHLH28	*rna-Par09411.1*	717	141	187	259	130	62.2	37.8
ParbHLH29	*rna-Par09510.1*	1029	331	207	236	255	43.05	56.95
ParbHLH30	*rna-Par09662.1*	684	166	149	219	150	53.8	46.2
ParbHLH31	*rna-Par11162.1*	750	198	183	198	171	50.8	49.2
ParbHLH32	*rna-Par11808.1*	975	271	237	196	271	44.41	55.59
ParbHLH33	*rna-Par12177.1*	864	237	219	185	223	46.76	53.24
ParbHLH34	*rna-Par12585.1*	888	233	206	227	222	48.76	51.24
ParbHLH35	*rna-Par12709.1*	561	185	94	143	139	42.25	57.75
ParbHLH36	*rna-Par13201.1*	3168	843	715	774	836	47	53
ParbHLH37	*rna-Par13621.1*	276	80	69	67	60	49.28	50.72
ParbHLH38	*rna-Par13827.1*	2973	870	611	719	773	44.74	55.26
ParbHLH39	*rna-Par14727.1*	873	268	193	194	218	44.33	55.67
ParbHLH40	*rna-Par15030.1*	675	237	136	152	150	42.67	57.33
ParbHLH41	*rna-Par15981.1*	1329	407	300	282	340	43.79	56.21
ParbHLH42	*rna-Par16654.1*	1290	393	252	306	339	43.26	56.74
ParbHLH43	*rna-Par17969.1*	1278	359	294	320	305	48.04	51.96
ParbHLH44	*rna-Par18724.1*	1533	390	303	426	414	47.55	52.45
ParbHLH45	*rna-Par18787.1*	1521	410	415	403	293	53.78	46.22
ParbHLH46	*rna-Par18834.1*	1296	408	315	281	292	45.99	54.01
ParbHLH47	*rna-Par19372.1*	1251	332	295	356	268	52.04	47.96
ParbHLH48	*rna-Par19701.1*	762	231	163	169	199	43.57	56.43
ParbHLH49	*rna-Par19702.1*	633	196	124	151	162	43.44	56.56
ParbHLH50	*rna-Par19923.1*	1170	322	339	222	287	47.95	52.05

ParbHLH51	*rna-Par20469.1*	2004	527	577	517	383	54.59	45.41
ParbHLH52	*rna-Par20574.1*	897	235	213	252	197	51.84	48.16
ParbHLH53	*rna-Par20622.1*	1500	428	260	404	408	44.27	55.73
ParbHLH54	*rna-Par20837.1*	1560	409	431	403	317	53.46	46.54
ParbHLH55	*rna-Par20994.1*	1827	575	421	389	442	44.33	55.67
ParbHLH56	*rna-Par21276.1*	1026	327	211	257	231	45.61	54.39
ParbHLH57	*rna-Par21277.1*	1368	456	262	317	333	42.32	57.68
ParbHLH58	*rna-Par21338.1*	1017	311	241	217	248	45.03	54.97
ParbHLH59	*rna-Par21369.1*	1035	329	217	229	260	43.09	56.91
ParbHLH60	*rna-Par21489.1*	1134	358	221	265	290	42.86	57.14
ParbHLH61	*rna-Par21490.1*	1074	355	203	252	264	42.36	57.64
ParbHLH62	*rna-Par21631.1*	939	278	196	230	235	45.37	54.63
ParbHLH63	*rna-Par23569.1*	2058	505	557	588	408	55.64	44.36
ParbHLH64	*rna-Par24331.1*	1911	599	345	467	500	42.49	57.51
ParbHLH65	*rna-Par24658.1*	1566	484	312	356	414	42.66	57.34
ParbHLH66	*rna-Par24659.1*	1197	343	245	292	317	44.86	55.14
ParbHLH67	*rna-Par24660.1*	1536	474	290	349	423	41.6	58.4
ParbHLH68	*rna-Par24662.1*	1452	433	301	349	369	44.77	55.23
ParbHLH69	*rna-Par24663.1*	1482	442	294	361	385	44.2	55.8
ParbHLH70	*rna-Par24782.1*	1626	425	340	421	440	46.8	53.2
ParbHLH71	*rna-Par24792.1*	1032	320	230	227	255	44.28	55.72
ParbHLH72	*rna-Par25080.1*	561	170	107	145	139	44.92	55.08
ParbHLH73	*rna-Par25081.1*	561	174	107	138	142	43.67	56.33

ParbHLH74	*rna-Par25162.1*	1107	333	222	299	253	47.06	52.94
ParbHLH75	*rna-Par25227.1*	279	79	57	75	68	47.31	52.69
ParbHLH76	*rna-Par25335.1*	1224	359	305	283	277	48.04	51.96
ParbHLH77	*rna-Par25535.1*	849	292	169	194	194	42.76	57.24
ParbHLH78	*rna-Par26161.1*	756	222	175	163	196	44.71	55.29
ParbHLH79	*rna-Par26258.1*	729	217	164	154	194	43.62	56.38
ParbHLH80	*rna-Par26259.1*	732	220	151	162	199	42.76	57.24
ParbHLH81	*rna-Par26262.1*	741	226	156	159	200	42.51	57.49
ParbHLH82	*rna-Par26267.1*	693	214	150	150	179	43.29	56.71
ParbHLH83	*rna-Par26657.1*	1032	280	297	255	200	53.49	46.51
ParbHLH84	*rna-Par26668.1*	972	283	219	227	243	45.88	54.12
ParbHLH85	*rna-Par27199.1*	744	240	179	150	175	44.22	55.78
ParbHLH86	*rna-Par27557.1*	981	286	249	208	238	46.59	53.41
ParbHLH87	*rna-Par27964.1*	1620	491	388	313	428	43.27	56.73
ParbHLH88	*rna-Par28318.1*	1113	250	379	284	200	59.57	40.43

Table S12 A comparison of composition, physical and chemical characters of *bHLH* genes

Gene name	Molecular formula	Molecular weight /Da	number	PI	negatively	positively	Protein instability index	Lipid binding index	The richest amino acid
ParbHLH1	$C_{1941}H_{3063}N_{587}O_{654}S_{21}$	45759.47	419	6.64	43	41	56.4	54.06	Ser
ParbHLH2	$C_{1591}H_{2504}N_{468}O_{494}S_{19}$	36701.36	328	6.2	37	31	49.32	75.52	Leu

ParbHLH3	$C_{1045}H_{1734}N_{310}O_{302}S_{10}$	23793.76	215	9.91	23	38	47.21	90.19	Val
ParbHLH4	$C_{1443}H_{2262}N_{400}O_{478}S_{15}$	33343.13	296	4.79	48	32	74.17	64.26	Ser
ParbHLH5	$C_{1750}H_{2818}N_{480}O_{573}S_{12}$	40135.22	363	6.42	46	44	54.01	76.56	Ser
ParbHLH6	$C_{2263}H_{3566}N_{624}O_{699}S_{20}$	51340.17	456	7.99	52	54	45.54	73.99	Ser
ParbHLH7	$C_{922}H_{1413}N_{267}O_{293}S_{8}$	21182.46	188	7.2	19	19	60.78	56.01	Ser
ParbHLH8	$C_{3384}H_{5420}N_{1016}O_{1137}S_{50}$	80133.39	746	6.06	78	69	53.01	60.76	Ser
ParbHLH9	$C_{1682}H_{2618}N_{476}O_{536}S_{17}$	38629.18	344	5.29	46	28	52.79	75.44	Leu
ParbHLH10	$C_{1547}H_{2459}N_{463}O_{492}S_{16}$	35929.31	341	5.74	35	28	54.12	71.55	Gly
ParbHLH11	$C_{2131}H_{3373}N_{611}O_{688}S_{21}$	49234.16	440	5.02	64	44	57.23	70.93	Glu
ParbHLH12	$C_{1029}H_{1659}N_{301}O_{305}S_{14}$	23576.17	202	9.32	27	32	43.78	73.91	Glu
ParbHLH13	$C_{1207}H_{1949}N_{327}O_{385}S_{9}$	27490.25	242	5.48	41	36	65.57	84.63	Glu
ParbHLH14	$C_{1011}H_{1655}N_{289}O_{303}S_{7}$	22931.44	205	8.69	20	23	67.67	104.49	Ser
ParbHLH15	$C_{2593}H_{4144}N_{768}O_{864}S_{24}$	60671.49	567	7.06	60	60	53.88	60.46	Ser
ParbHLH16	$C_{1218}H_{1959}N_{365}O_{405}S_{12}$	28580.88	263	6.71	31	30	42.56	67.83	Ser
ParbHLH17	$C_{1912}H_{3101}N_{541}O_{590}S_{12}$	43492.64	389	8.7	47	51	55.36	88.2	Leu
ParbHLH18	$C_{1664}H_{2692}N_{508}O_{513}S_{13}$	38439.56	353	9.45	36	47	57.47	76.49	Ser
ParbHLH19	$C_{443}H_{757}N_{143}O_{154}S_{4}$	10678.99	94	7.93	14	15	82.98	91.28	Ser
ParbHLH20	$C_{1576}H_{2504}N_{474}O_{528}S_{8}$	36796.56	335	5.83	44	37	54.07	65.52	Ser
ParbHLH21	$C_{2566}H_{4075}N_{751}O_{857}S_{26}$	59991.66	561	5.44	66	53	53.14	61.89	Ser
ParbHLH22	$C_{1752}H_{2800}N_{520}O_{554}S_{14}$	40461.5	360	6.25	42	35	69.17	75.11	Gln
ParbHLH23	$C_{2441}H_{3831}N_{695}O_{744}S_{32}$	55844.4	504	8.77	37	43	57.85	65.81	Ser
ParbHLH24	$C_{1994}H_{3137}N_{581}O_{668}S_{20}$	46578.53	432	5.62	50	40	59.64	60.72	Ser
ParbHLH25	$C_{1811}H_{2820}N_{520}O_{613}S_{16}$	42198.39	382	5.16	55	38	52.81	61.28	Ser

ParbHLH26	$C_{1159}H_{1864}N_{340}O_{369}S_{17}$	27010.63	244	9.3	20	25	59.32	64.39	Ser
ParbHLH27	$C_{1564}H_{2470}N_{456}O_{492}S_{16}$	36046.54	321	6.62	35	34	73.39	69.88	Ser
ParbHLH28	$C_{1111}H_{1807}N_{353}O_{356}S_{11}$	26158.38	238	8.63	31	34	50.34	73.03	Arg
ParbHLH29	$C_{1671}H_{2617}N_{461}O_{551}S_{23}$	38718.3	342	4.49	53	30	58.26	70.44	Leu
ParbHLH30	$C_{1046}H_{1682}N_{314}O_{346}S_{9}$	24481.3	227	5.57	32	28	52.06	69.25	Ser
ParbHLH31	$C_{1206}H_{1931}N_{341}O_{369}S_{8}$	27368.14	249	7.69	32	33	47.36	77.95	Leu
ParbHLH32	$C_{1658}H_{2529}N_{415}O_{495}S_{13}$	36612.58	324	5.03	41	27	53.74	78.33	Leu
ParbHLH33	$C_{1429}H_{2221}N_{373}O_{447}S_{15}$	32259.49	287	4.78	33	23	66.43	75.16	Leu
ParbHLH34	$C_{1385}H_{2239}N_{395}O_{441}S_{8}$	31736.88	295	8.79	35	39	47.4	79.73	Ser
ParbHLH35	$C_{895}H_{1463}N_{261}O_{281}S_{10}$	20696.64	186	7.78	24	25	41.78	82.8	Ser
ParbHLH36	$C_{5102}H_{8156}N_{1436}O_{1581}S_{46}$	116384.31	1055	6.34	122	116	49.14	84.56	Leu
ParbHLH37	$C_{439}H_{751}N_{137}O_{147}S_{2}$	10364.74	91	7.93	14	15	80.6	101.76	Ser
ParbHLH38	$C_{4919}H_{7712}N_{1412}O_{1478}S_{34}$	111369.96	990	7.11	118	116	53.95	78.38	Ser
ParbHLH39	$C_{1412}H_{2190}N_{402}O_{459}S_{11}$	32494	290	6.31	37	33	43.34	58.17	Ser
ParbHLH40	$C_{1132}H_{1804}N_{328}O_{356}S_{8}$	25961.24	224	6.15	34	30	55.91	78.75	Ser
ParbHLH41	$C_{3253}H_{5155}N_{967}O_{1045}S_{27}$	75397.19	672	6.48	80	73	53.34	70.67	Ser
ParbHLH42	$C_{2048}H_{3211}N_{627}O_{666}S_{26}$	48106.38	429	8.07	46	48	53.78	55.06	Ser
ParbHLH43	$C_{2065}H_{3260}N_{586}O_{649}S_{12}$	47064.86	425	5.81	54	44	50.06	80.73	Ser
ParbHLH44	$C_{2431}H_{3872}N_{702}O_{781}S_{19}$	56038.86	510	5.59	66	57	47.6	77.43	Ser
ParbHLH45	$C_{2454}H_{3876}N_{706}O_{781}S_{18}$	56343.11	506	5.67	69	55	42.3	77.45	Ser
ParbHLH46	$C_{2077}H_{3329}N_{611}O_{675}S_{24}$	48429.41	431	5.6	58	45	67.52	72.44	Ser
ParbHLH47	$C_{1965}H_{3180}N_{572}O_{631}S_{23}$	45651.7	416	5.8	59	53	48	79.81	Ala
ParbHLH48	$C_{1212}H_{2004}N_{374}O_{373}S_{15}$	28264.43	253	9.55	26	38	58.12	83.6	Ser

ParbHLH49	$C_{1004}H_{1666}N_{298}O_{315}S_{8}$	23208.56	210	9.66	20	28	58.12	92.33	Ser
ParbHLH50	$C_{1853}H_{2929}N_{505}O_{601}S_{14}$	42346.5	389	5.01	42	30	67.38	74.99	Ser
ParbHLH51	$C_{3234}H_{5115}N_{921}O_{1048}S_{21}$	74339.99	667	5.05	99	67	71.41	72.94	Ser
ParbHLH52	$C_{1373}H_{2209}N_{407}O_{431}S_{13}$	31730.89	298	6.01	34	31	48.24	77.32	Ala
ParbHLH53	$C_{2430}H_{3826}N_{676}O_{762}S_{20}$	55344.38	499	5.42	68	55	49.73	76.49	Ser
ParbHLH54	$C_{2413}H_{3832}N_{720}O_{787}S_{38}$	56739.6	519	6.09	57	51	64.26	51.77	Ser
ParbHLH55	$C_{2879}H_{4559}N_{839}O_{939}S_{27}$	66815.55	608	6.72	62	60	48.91	64.85	Ser
ParbHLH56	$C_{1648}H_{2572}N_{498}O_{547}S_{17}$	38658.58	341	5.06	63	34	54.03	67.8	Asp
ParbHLH57	$C_{2181}H_{3416}N_{634}O_{737}S_{19}$	50920.06	455	4.74	70	40	46.76	69.36	Asn
ParbHLH58	$C_{1614}H_{2589}N_{477}O_{529}S_{16}$	37653.15	338	7.06	44	44	65.96	57.66	Ser
ParbHLH59	$C_{1670}H_{2676}N_{464}O_{526}S_{20}$	38311.61	344	7.05	40	40	44.5	76.51	Ser
ParbHLH60	$C_{1752}H_{2782}N_{502}O_{607}S_{22}$	41295.68	377	5.06	46	32	59.57	67.51	Ser
ParbHLH61	$C_{1691}H_{2637}N_{491}O_{587}S_{17}$	39782.5	357	4.94	51	32	52.75	61.48	Ser
ParbHLH62	$C_{1462}H_{2333}N_{419}O_{458}S_{17}$	33653.16	312	8.45	29	32	45.79	72.88	Gly
ParbHLH63	$C_{3238}H_{5116}N_{922}O_{1070}S_{20}$	74722.98	685	5.44	83	70	48.62	69.23	Ser
ParbHLH64	$C_{3096}H_{4953}N_{883}O_{978}S_{33}$	71251.69	636	5.75	86	75	52.57	82.58	Ser
ParbHLH65	$C_{2566}H_{4049}N_{753}O_{787}S_{20}$	58681.15	521	8.61	60	65	51.46	76.53	Ser
ParbHLH66	$C_{1976}H_{3077}N_{557}O_{598}S_{20}$	44845.74	398	6.8	46	45	47.77	74.97	Ser
ParbHLH67	$C_{2502}H_{3895}N_{715}O_{785}S_{18}$	57128.85	511	6.07	63	54	44.06	76.26	Ser
ParbHLH68	$C_{2362}H_{3691}N_{683}O_{744}S_{18}$	54137.5	483	6.33	57	52	49.31	76.25	Ser
ParbHLH69	$C_{2419}H_{3784}N_{714}O_{747}S_{17}$	55366.01	493	7.06	58	57	53.32	73.35	Ser
ParbHLH70	$C_{2535}H_{4039}N_{741}O_{827}S_{22}$	58834.74	541	5.43	67	55	46.17	76.04	Leu

ParbHLH71	$C_{1662}H_{2641}N_{473}O_{539}S_{13}$	38289.88	343	6.67	43	42	52.23	66.79	Ser
ParbHLH72	$C_{910}H_{1510}N_{266}O_{282}S_{9}$	20978.15	186	9.25	23	28	46.8	93.23	Ser
ParbHLH73	$C_{917}H_{1514}N_{264}O_{285}S_{7}$	21022.13	186	9.16	24	28	39.97	93.76	Ser
ParbHLH74	$C_{1715}H_{2760}N_{502}O_{562}S_{16}$	39916.77	368	6.12	48	44	52.35	71.85	Ser
ParbHLH75	$C_{427}H_{730}N_{142}O_{148}S_{2}$	10285.48	92	9.17	12	14	95.36	93.37	Ser
ParbHLH76	$C_{1882}H_{2991}N_{585}O_{597}S_{32}$	44390.93	407	8.41	34	36	59.86	55.7	Ser
ParbHLH77	$C_{1372}H_{2127}N_{397}O_{444}S_{15}$	31768.28	282	5.7	37	31	48.49	56.35	Ser
ParbHLH78	$C_{1219}H_{1984}N_{362}O_{391}S_{8}$	28223.83	251	7.05	34	34	51.27	88.21	Leu
ParbHLH79	$C_{1188}H_{1921}N_{341}O_{377}S_{8}$	27269.86	242	6.71	29	28	70.52	92.73	Ser
ParbHLH80	$C_{1212}H_{1947}N_{335}O_{391}S_{6}$	27660.16	243	5.54	34	29	64.4	89.09	Ser
ParbHLH81	$C_{1242}H_{1988}N_{344}O_{389}S_{6}$	28155.88	246	6.34	32	30	66.66	88.37	Ser
ParbHLH82	$C_{1151}H_{1856}N_{326}O_{359}S_{10}$	26325.97	230	6.61	28	27	69.13	89.39	Leu
ParbHLH83	$C_{1646}H_{2627}N_{477}O_{521}S_{11}$	37787.51	343	5.97	43	37	50.92	73.73	Ser
ParbHLH84	$C_{1555}H_{2465}N_{479}O_{498}S_{13}$	36255.37	323	9	33	38	49.98	64.92	Ser
ParbHLH85	$C_{1203}H_{1929}N_{355}O_{373}S_{21}$	28006.96	247	7.96	26	27	58.22	65.18	Ser
ParbHLH86	$C_{1528}H_{2442}N_{458}O_{520}S_{12}$	35933.68	326	6.02	43	40	61.56	56.6	Ser
ParbHLH87	$C_{2616}H_{4209}N_{767}O_{823}S_{19}$	60182.98	539	8.84	52	57	71.64	80.72	Ser
ParbHLH88	$C_{1601}H_{2568}N_{494}O_{554}S_{22}$	38306.3	370	6.13	32	28	51.05	59.46	Ser

Table S13. Estimated Ka/Ks ratios of the duplicated *bHLH* gene in apricot

No.	Paralogous pairs	Ka[a]	Ks[b]	Ka/Ks	Effective length /bp	Average S-sites[c]	Average N-sites[d]

1	*ParbHLH5/ ParbHLH6*	0.281363492	1.000286101	0.281283017	1044	233.08	810.92
2	*ParbHLH48/ ParbHLH49*	0.386998333	1.809148348	0.213911885	615	144.75	470.25
3	*ParbHLH56/ ParbHLH57*	0.268478877	1.097639648	0.244596556	804	164.08	639.92
4	*ParbHLH60/ ParbHLH61*	0.374446430	0.881357734	0.424851812	1026	217.17	808.83
5	*ParbHLH65/ ParbHLH66*	0.116463832	0.382044719	0.304843456	1173	258.33	914.67
6	*ParbHLH66/ ParbHLH67*	0.098366428	0.402020753	0.244679975	1158	252.25	905.75
7	*ParbHLH68/ ParbHLH69*	0.153537030	0.663560427	0.231383645	1431	319.42	1111.58
8	*ParbHLH72/ ParbHLH73*	0.025723488	0.016439014	1.564782864	558	123.00	435.00
9	*ParbHLH79/ ParbHLH80*	0.095936126	0.242009186	0.396415225	726	168.00	558.00

Note: A non-synonymous site substitution rate; b synonymous site substitution rate; c average number of synonymous sites; d average number of non-synonymous sites

Table S14 Addresses of 15 species genomes

Species	Website
P. armeniaca	Prunus armeniaca (ID 577047) - BioProject - NCBI (nih.gov)
P. mume	https://www.ncbi.nlm.nih.gov/bioproject/?term=PRJNA352648
P. persica	https://www.ncbi.nlm.nih.gov/nuccore/AKXU00000000.1
P. avium	https://www.ncbi.nlm.nih.gov/biosample/SAMN13640536/
P. salicina	https://www.ncbi.nlm.nih.gov/search/all/?term=WERZ00000000
M. domestica	https://www.ncbi.nlm.nih.gov/bioproject/PRJNA482033/
P. bretschneideri	https://www.ncbi.nlm.nih.gov/nuccore/AJSU01000000
E. japonica	https://ftp.cngb.org/pub/CNSA/data3/CNP0001531/
G. trifoliata	https://ftp.cngb.org/pub/CNSA/data3/CNP0001531/
R. rugosa	http://eplantftp.njau.edu.cn/Rosa_rugosa/
R. occidentalis	https://www.rosaceae.org/analysis/268

F. vesca	https://www.rosaceae.org/species/fragaria_vesca/genome_v4.0.a2
P. micrantha	https://www.rosaceae.org/analysis/274
A. chinensis	https://figshare.com/articles/dataset/Actinidia_chinensis_genome_data/10046558
Z. jujuba	https://www.ncbi.nlm.nih.gov/search/all/?term=+JREP00000000
