# Supplementary figures and images for: A Chromosome-Level Genome of ‘Xiaobaixing’ (Prunus armeniaca L.) Provides Clues to Its Domestication and Identification of Key bHLH Genes in Amygdalin Biosynthesis
Source: Plants (Basel). 2023 Jul 25;12(15):2756. doi: 10.3390/plants12152756 (PMC10421183; doi:10.3390/plants12152756)

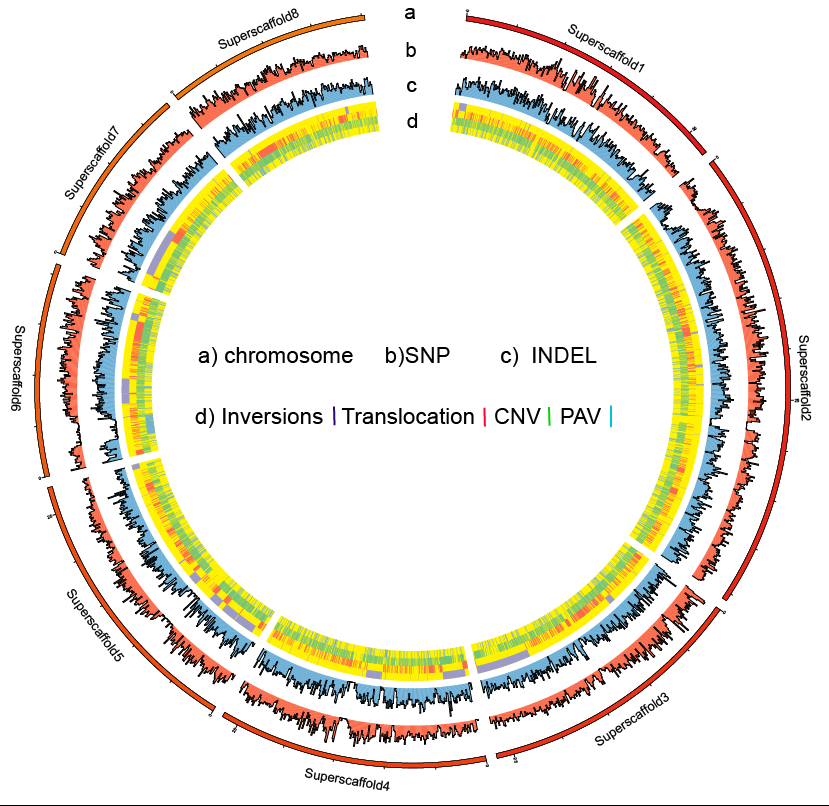

Supplement: Supplementary file 1 [file plants-12-02756-s001.zip › Figure S1.png]

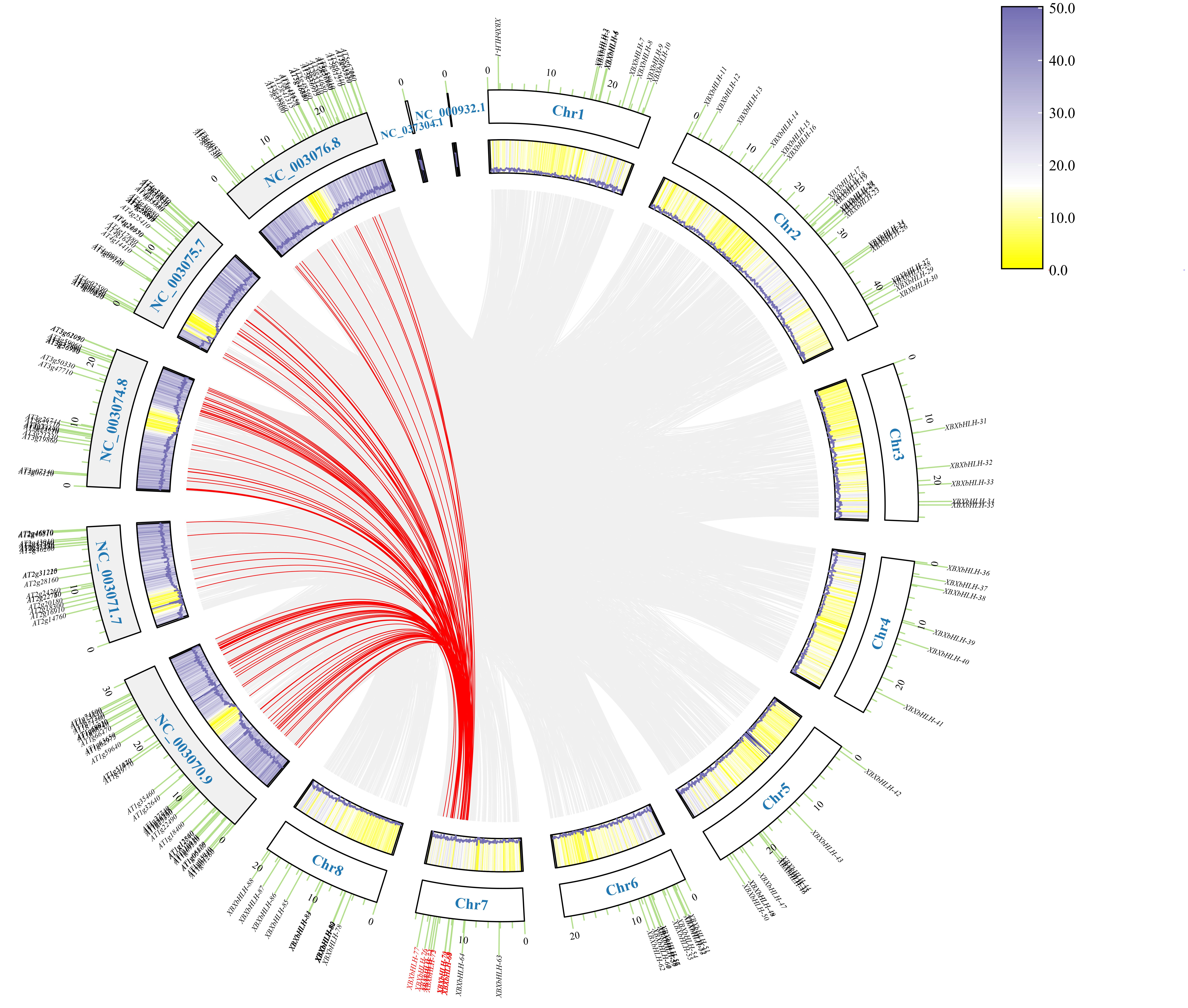

Supplement: Supplementary file 1 [file plants-12-02756-s001.zip › Figure S2.jpg]
